# Dangers of *Clostridium perfringens* food poisoning in psychiatric patients

**DOI:** 10.4102/sajpsychiatry.v25i0.1339

**Published:** 2019-08-26

**Authors:** Colleen Bamford, Peter Milligan, Sean Kaliski

**Affiliations:** 1 Medical Microbiologist, Pathcare, East London, South Africa; 2 National Health Laboratory Service, Groote Schuur Hospital, Cape Town, South Africa; 3 Division of Medical Microbiology, Department of Pathology, University of Cape Town, Cape Town, South Africa; 4 Department of Psychiatry, Ngwelezana Hospital, Empangeni, South Africa; 5 Forensic Mental Health Service, Valkenberg Hospital, Cape Town, South Afria; 6 Department of Psychiatry and Mental Health, University of Cape Town, Cape Town, South Africa

**Keywords:** *Clostridium perfringens*, Food poisoning, Psychiatric patients, Fatal, Gastric hypomotility, Clozapine

## Abstract

*Clostridium perfringens* food poisoning can be fatal in patients with chronic constipation. We report the investigation and management of a probable outbreak of *C. perfringens* food poisoning among psychiatric patients in Cape Town, South Africa, in 2013.

The two major public health events in South Africa in the past few years – namely, the listeriosis outbreak and the Life Esidimeni saga – have highlighted the critical role of food safety and the dangers of foodborne illnesses on the one hand, and the vulnerability of persons with severe mental illness and disability on the other hand. Another food-related disease, *Clostridium perfringens* food poisoning, usually associated only with a self-limiting gastrointestinal illness, may be fatal in this vulnerable patient population. We share our experience of the investigation and management of a probable outbreak of *C. perfringens* food poisoning that occurred in Cape Town in January 2013.^1^

On a single day in midsummer, three in-patients at a state psychiatric hospital experienced the acute onset of vomiting and abdominal pain with distension, which was followed within hours by sudden collapse and death. Post-mortem examination revealed only massive dilatation of the colon in all. Seven additional patients were reported with symptoms of diarrhoea or vomiting on the same day. None required specific treatment and all resolved spontaneously. *C. perfringens* type A enterotoxin was detected in the stools of the three patients who died and of three patients with diarrhoea.

The only common exposure between the three fatal cases was food from the hospital’s central kitchen. Although the evening meal of the preceding day was considered suspicious, there was unfortunately no left-over food available for testing for presence of *C. perfringens* toxins. An inspection of the hospital kitchen a few days later by a regional government environmental health practitioner confirmed that the kitchen complied with all relevant health and environmental health legislation, including safe preparation and storage of foods. However, subsequent investigation showed that temperature of the food was not adequately controlled after leaving the kitchen for distribution to the wards. There was a delay of approximately 3 hours between dispatch of food from the kitchen and serving of the meal with daytime temperatures of 30 °C at that time of the year. *Clostridium perfringens*, a spore-forming organism, may survive normal cooking temperatures, and if not served or refrigerated immediately, surviving spores may germinate in cooked food and produce toxins.

While fatalities due to Enterotoxigenic *C. perfringens* type A^2^ are rare, unexpected deaths^3^ and necrotising colitis^4,5^ have been reported in patients with chronic constipation, which may be related to use of psychotropic medications with anticholinergic side effects. It is presumed that the delayed emptying of the gastrointestinal tract allows for increased absorption of the toxin. Clozapine in particular has been noted to be associated with a relatively high prevalence of serious gastric hypomotility, of the order of three cases per 1000 patients exposed.^6,7^ Two of the patients were on clozapine and the third was on amitryptiline which is frequently associated with constipation. However, the relationship between these drugs and severe *C. perfringens* disease is uncertain, since many patients receiving these commonly prescribed medications did not develop disease, both in this and previously reported outbreaks. Additional factors, such as the amount of toxin ingested, and other factors may influence the development and severity of subsequent disease.

Although not proven, due to the non-availability of food samples and incomplete testing, this fatal outbreak was likely caused by *C. perfringens* food poisoning. Similar cases may not be recognised, particularly if they are sporadic or occur in the community setting. This condition is preventable by the institution of measures to ensure adequate temperature control of pre-prepared food. Vigilance regarding food safety is especially required in psychiatric facilities and for persons in the community taking medications leading to gastric hypomotility. Clinicians should be aware of this potentially fatal condition and should actively prevent and manage constipation in these patients.
