# Corrigendum: Dangers of *Clostridium perfringens* food poisoning in psychiatric patients

**DOI:** 10.4102/sajpsychiatry.v27i0.1771

**Published:** 2021-10-15

**Authors:** Colleen Bamford, Peter Milligan, Sean Kaliski

**Affiliations:** 1 National Health Laboratory Service, Groote Schuur Hospital, Cape Town, South Africa; 2 Division of Medical Microbiology, Department of Pathology, University of Cape Town, Cape Town, South Africa; 3 Acute services, Valkenberg Hospital, Cape Town, South Africa; 4 Department of Psychiatry and Mental Health, University of Cape Town, Cape Town, South Africa; 5 Forensic Mental Health Service, Valkenberg Hospital, Cape Town, South Africa

In the published article, Bamford C, Milligan P, Kaliski S. Dangers of *Clostridium perfringens* food poisoning in psychiatric patients. S Afr J Psychiatr. 2019;25(0):a1339. https://doi.org/10.4102/sajpsychiatry.v25i0.1339, there was an error in the affiliations. Instead of:

**‘Authors:**

Colleen Bamford^1,2,3^

Peter Milligan^4,6^

Sean Kaliski^5,6^

**Affiliations:**

^1^Medical Microbiologist, Pathcare, East London, South Africa

^2^National Health Laboratory Service, Groote Schuur Hospital, Cape Town, South Africa

^3^Division of Medical Microbiology, Department of Pathology, University of Cape Town, Cape Town, South Africa

^4^Department of Psychiatry, Ngwelezana Hospital, Empangeni, South Africa

^5^Forensic Mental Health Service, Valkenberg Hospital, Cape Town, South Afria

^6^Department of Psychiatry and Mental Health, University of Cape Town, Cape Town, South Africa’,

It should be:

**‘Authors:**

Colleen Bamford^1,2^

Peter Milligan^3,4^

Sean Kaliski^4,5^

**Affiliations:**

^1^National Health Laboratory Service, Groote Schuur Hospital, Cape Town, South Africa

^2^Division of Medical Microbiology, Department of Pathology, University of Cape Town, Cape Town, South Africa

^3^Acute services, Valkenberg Hospital, Cape Town, South Africa

^4^Department of Psychiatry and Mental Health, University of Cape Town, Cape Town, South Africa

^5^Forensic Mental Health Service, Valkenberg Hospital, Cape Town, South Africa’.

The authors apologise for this error. The correction does not change the study’s findings of significance or overall interpretation of the study’s results or the scientific conclusions of the article in any way.

